# Early Urine Output in the Emergency Room as a Prognostic Indicator for Critically Ill Patients Undergoing Continuous Renal Replacement

**DOI:** 10.3390/life15060866

**Published:** 2025-05-27

**Authors:** Soo Hyun Han, Changshin Kang, Hyerim Park, Eu Jin Lee, Young Rok Ham, Ki Ryang Na, Jung Soo Park, Dae Eun Choi

**Affiliations:** 1 Department of Nephrology, College of Medicine, Chungnam National University, 266 Munwha-ro, Jung-gu, Daejeon 35015, Republic of Korea; medihsh77@gmail.com (S.H.H.); eujinlee@cnuh.co.kr (E.J.L.); youngrok01@cnuh.co.kr (Y.R.H.); drngr@cnu.ac.kr (K.R.N.); 2 Department of Emergency Medicine, Chungnam National University Hospital, 282 Munwha-ro, Jung-gu, Daejeon 35015, Republic of Korea; rosc@cnu.ac.kr; 3 Department of Medical Sciences, College of Medicine, Chungnam National University, 266 Munwha-ro, Jung-gu, Daejeon 35015, Republic of Korea; hye05240@gmail.com

**Keywords:** acute kidney injury, urine output, 30-day mortality, 90-day mortality, emergency room

## Abstract

Objectives: The impact of initial emergency room (ER) factors on survival and renal function in critically ill patients undergoing continuous renal replacement therapy (CRRT) remains unclear. This study aimed to evaluate whether these initial factors influence survival and renal recovery in such patients. Methods: This single-center, retrospective study included 190 critically ill patients admitted to the intensive care unit (ICU) via the ER for CRRT between 1 March 2018, and 31 May 2021. Clinical parameters, including urine output, estimated glomerular filtration rate (eGFR), and serum neutrophil gelatinase-associated lipocalin (NGAL), were assessed. The primary outcomes were 30-day and 90-day mortality, while secondary outcomes included 30-day and 90-day RRT-free durations. Results: Patients with low urine output (LUO, defined as the average of <0.5 mL/kg/h over 6 h) were significantly associated with higher 30-day and 90-day mortality rates. Multivariable Cox regression analysis revealed that the LUO group had an increased risk of 30-day and 90-day mortality (hazard ratios: 1.935 and 2.141, respectively) compared to the high urine output (HUO, defined as the average of ≥0.5 mL/kg/h over 6 h) group. No significant association was observed between mortality and initial eGFR or plasma NGAL levels. However, the HUO group and patients with initial eGFR ≥ 30 mL/min/1.73 m^2^ had longer RRT-free durations at 30 and 90 days. Plasma NGAL levels did not significantly correlate with RRT-free durations. Conclusions: Initial 6-h urine output in the ER is a significant predictor of 30-day and 90-day mortality in critically ill patients undergoing CRRT.

## 1. Introduction

Acute kidney injury (AKI) is a common complication that significantly worsens outcomes in hospitalized patients [1]. The clinical spectrum of AKI ranges from minor alterations in kidney function to severe renal dysfunction requiring renal replacement therapy (RRT) [2]. Furthermore, AKI is associated with long-term adverse outcomes, including an increased risk of chronic kidney disease and mortality [3,4,5]. Among ICU patients, those with severe AKI requiring RRT experience a mortality rate exceeding 50% [6,7,8].

Numerous studies have explored factors influencing survival in AKI patients undergoing RRT in the ICU. Risk prediction tools such as the Acute Physiology and Chronic Health Evaluation II (APACHE II), Sequential Organ Failure Assessment (SOFA), and Simplified Acute Physiology Score II (SAPS II) include AKI as a significant risk factor for critically ill patients [9,10,11]. Decreased urine output and elevated serum creatinine (sCr) are particularly critical indicators of AKI in the ICU setting [12]. Urine output, in particular, is a simple yet crucial factor closely linked to survival in critically ill patients [13,14]. For instance, Heffernan et al. [15] identified reduced urine output as a predictor of mortality in ICU patients, while Lee et al. [16] reported that urine output during the first day of CRRT is the strongest risk factor for in-hospital mortality.

Early management of volume status, blood pressure, acid–base balance, and electrolyte levels is essential for critically ill patients [17,18]. By the time ICU patients receiving CRRT are admitted via the emergency room (ER), their condition is often already critical. Thus, early identification and management of AKI in the ER are vital. While previous studies have primarily focused on clinical factors affecting outcomes after ICU admission, there is limited research on the impact of initial clinical factors in the ER on mortality and hospitalization in critically ill patients [15,16]. Identifying fast and simple predictors of mortality in the ER could enable earlier intervention and improve patient outcomes.

This study aimed to investigate which initial clinical factors in the ER are critical for mortality in critically ill patients admitted to the ICU and undergoing CRRT.

## 2. Methods

### 2.1. Research Design

The study is a single-center retrospective chart review consisting of patients admitted to ICUs through ERs of Chungnam National University Hospital, Daejeon, South Korea, between 1 March 2018, and 31 May 2021. The Institutional Review Board of Chungnam National University Hospital (No. 2021-11-029) approved this study. We reviewed the medical records of patients who were treated with CRRT among ICU patients admitted via ER. The following data were collected based on when the patient arrived at the emergency room. Baseline demographics like age, sex, systolic blood pressure, diastolic blood pressure, and body mass index (BMI) were collected. Further, existing comorbidities and laboratory findings were recorded. We evaluated hourly urine output using an inserted foley catheter and the initial urine volume collected as soon as insertion was performed; but residual volume was excluded. Additionally, we classified the patients into two groups based on a urine output threshold of 0.5 mL/kg/h. Those with urine output less than 0.5 mL/kg/h over 6 h were assigned to the low urine output (LUO) group, while those with urine output equal to or greater than 0.5 mL/kg/h over the same period were assigned to the high urine output (HUO) group. The participants were categorized into three groups based on their initial estimated glomerular filtration rate (eGFR): those with eGFR < 15 mL/min/1.73 m^2^, those with eGFR < 30 mL/min/1.73 m^2^, and those with eGFR < 60 mL/min/m^2^.

### 2.2. Inclusion and Exclusion Criteria

Patients who were admitted to ICUs for CRRT through ERs were enrolled in the study. Chungnam National University Hospital has 4 intensive care units (ICUs) with 10 to 16 beds per ward and 4 emergency room (ER) units with 10 to 12 beds per ward. Study subjects were patients who were admitted to the ICU for RRT due to decreased urine output caused by various conditions such as sepsis or heart failure. Exclusion criteria were as follows: (1) patients aged <18 years; (2) those who had chronic kidney disease (CKD) stage IV or V (CKD staging was recognized prior to ICU admission); (3) those who had undergone renal replacement therapy, including peritoneal dialysis or hemodialysis; and (4) those approved for Physician Order for Life-Sustaining Treatment (POLST): the next generation in end-of-life planning for certain patients who wish to exercise prospective control over their own medical treatment in their final days [19].

### 2.3. Definitions

The AKI diagnostic criteria conformed to the 2012 KDIGO Clinical Practice Guidelines for AKI [20]: (1) increase in sCr > 0.3 mg/dL within 48 h, (2) increase in sCr > 1.5 times the baseline, or (3) the average urine volume of <0.5 mL/kg/h over 6 h. The low urine output (LUO) group was defined as the average urine volume of <0.5 mL/kg/h over 6 h. The corresponding cutoff for plasma neutrophil gelatinase-associated lipocalin (NGAL) was ≥364 ng/mL with 44% sensitivity for serious AKIs that required renal replacement therapy [21].

### 2.4. Initiation of CRRT

CRRT is initiated by the decision of a nephrologist for the following reasons: refractory pulmonary edema due to volume overload not responding to diuretics, refractory hyperkalemia, refractory metabolic acidosis, and symptomatic uremia [22]. Even though urine volume was sustained, some patients underwent early CRRT based on the clinical judgement of the nephrologist.

### 2.5. Statistical Analysis

Continuous variables were presented as mean ± standard deviation, and categorical variables were presented as absolute frequencies (percentage). Significant risk factors for critically ill patients’ mortality were identified using the Kaplan–Meier survival curve and Cox regression with 95% confidence intervals (CI). Student’s *t*-test and Pearson correlation were used as appropriate.

IBM SPSS Statistics 24.0 for Windows (IBM Corp., Armonk, NY, USA) was used for statistical analysis, and a *p*-value of <0.05 was considered statistically significant.

## 3. Results

### 3.1. Baseline Characteristics

**Figure 1:**
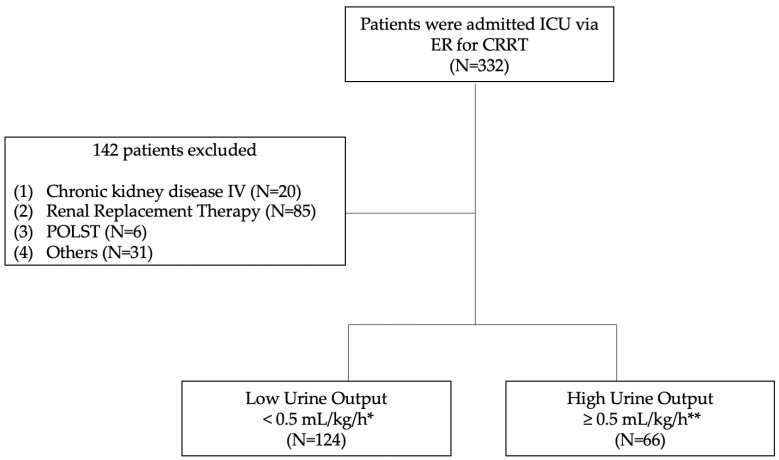
Flow diagram for patients. POLST (Physician Order for Life-Sustaining Treatment); * average urine volume of <0.5 mL/kg/h over 6 h; ** average urine volume of ≥0.5 mL/kg/h over 6 h.

Between March 2018 and May 2021, 332 patients were admitted to the ICU for CRRT through the ER. A total of 190 patients were included in the study after applying the exclusion criteria. The 190 patients were divided into two groups: the LUO group and the HUO group (Figure 1). No difference was observed in age and BMI between two groups (the LUO group and the HUO group). The HUO group had a higher mean systolic blood pressure and diastolic blood pressure. In laboratory parameters, the LUO group had a higher lactic acid level, NGAL, BUN, and serum creatinine than the HUO group. In addition, CRRT initiation occurred earlier in the LUO group than in the HUO group (mean 14.32 ± 3.79 h vs. 17.35 ± 5.50 h, respectively; *p* < 0.001), suggesting that lower urine output may prompt more rapid clinical decision-making for initiating CRRT. Other factors like hemoglobin, glucose, serum albumin, C-reactive protein, potassium, and volume of input fluid during the initial 6 hr were not statistically different between the HUO and LUO groups. A difference between the two groups was only observed in the following characteristic: cause of CRRT due to hypovolemic shock (Table 1).

### 3.2. 30-Day and 90-Day Mortality

**Figure 2:**
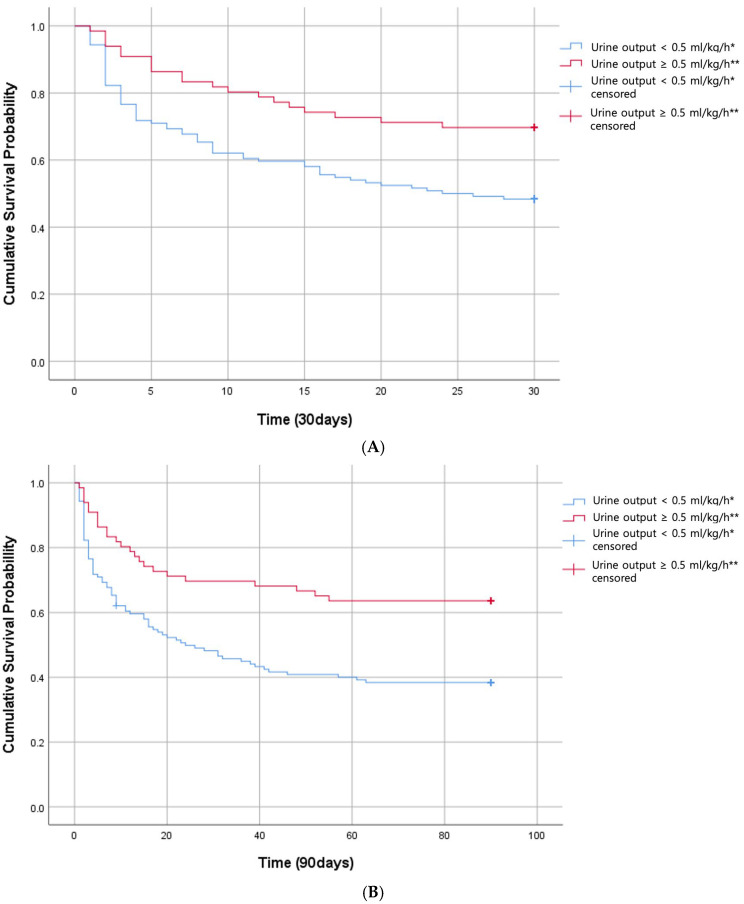
Primary outcome. Kaplan–Meier curves for cumulative probability of 30-day (**A**) and 90-day mortality (**B**). Low urine output (LUO) group demonstrates higher mortality compared with high urine output (HUO) group (*p =* 0.004 for 30-day mortality (**A**), and *p =* 0.001 for 90-day mortality (**B**)). * Average urine volume of <0.5 mL/kg/h over 6 h. ** Average urine volume of ≥0.5 mL/kg/h over 6 h. Urine output < 0.5 mL/kg/h: LUO (*n* = 124), urine output > 0.5 mL/kg/h: HUO (*n* = 66).

In terms of Kaplan–Meier curves for cumulative probability of 30-day mortality, the LUO group was significantly related to higher mortality (*p* = 0.004) (Figure 2). Cox regression analysis also resulted in higher mortality in the LUO group than in the HUO group. (hazard ratio [HR], 1.88; 95% CI, 1.10–3.25; *p* = 0.023) Additionally, a higher SOFA score was significantly associated with increased 30-day mortality (HR, 1.26; 95% CI, 1.09–1.46; *p* = 0.002) (Table 2). There was no significant difference between the groups divided by initial estimated GFR < 15 mL/min/1.73 m^2^, <30 mL/min/1.73 m^2^, and <60 mL/min/1.73 m^2^ (*p* = 0.999, *p* = 0.615, and *p* = 0.405, respectively). There was no significant difference in mortality in the two groups divided by plasma NGAL 364 ng/mL (*p* = 0.814, Figure S1). Furthermore, CRRT start time did not differ significantly between 30-day survivors and non-survivors (mean 15.26 h vs. 15.51 h, *p* = 0.721).

Kaplan–Meier curves for the cumulative probability of 90-day mortality showed a significantly higher risk in the LUO group than in the HUO group (*p* = 0.001) (Figure 2). Multivariable Cox regression analysis revealed that the LUO group was associated with a significant increase in mortality risk compared to the HUO group (HR, 2.07; 95% CI, 1.26–3.42; *p* = 0.017). In addition, SOFA score was also a significant independent predictor of 90-day mortality (HR, 1.21; 95% CI, 1.07–1.37; *p* = 0.003) (Table 2). There was no significant difference between the groups divided by initial estimated GFR < 15 mL/min/1.73 m^2^, <30 mL/min/1.73 m^2^, and <60 mL/min/1.73 m^2^ (*p* = 0.756, *p* = 0.247, and *p* = 0.372, respectively). There was no significant difference in 90-day mortality in the two groups divided by plasma NGAL 364 ng/mL (*p* = 0.937, Figure S2). Similarly, CRRT start time was not significantly associated with 90-day mortality (mean 15.43 h in survivors vs. 15.32 h in non-survivors, *p* = 0.868).

### 3.3. RRT-Free Days

RRT-free days were defined as the number of days excluding the period during which the patient received CRRT, as well as any subsequent period of intermittent hemodialysis following CRRT discontinuation. The HUO group was significantly associated with a higher number of RRT-free days through day 30 than the LUO group. The HUO group was also associated with a higher number of RRT-free days through day 90 than the LUO group (Table 3). By classifying initial eGFR results into three groups, it was observed that the group with initial eGFR < 15 mL/min/1.73 m^2^ had no significant relationship with RRT-free days through day 30 and day 90 (*p* = 0.056, *p* = 0.131). But the group with initial eGFR ≥ 30 mL/min/1.73 m^2^ was significantly associated with a higher number of RRT-free days through day 30 and day 90 (all *p* < 0.05).

## 4. Discussion

Our study demonstrated that urine volume in the first 6 h following arrival at the ER is a crucial factor in the survival of critically ill patients undergoing CRRT.

Various factors, including high SOFA score, mechanical ventilation and vasopressor use, and low serum albumin level, have been recognized as demonstrating an association with increased mortality in CRRT patients [23,24]. It has been also reported that urinary output is related to the survival rate of patients undergoing CRRT [25]. Cho et al. reported that the first-day urine output of patients undergoing CRRT is associated with 28-day mortality in sepsis-induced AKI patients [25]. Perez-Fernandez et al. reported that urine output during the 24 h prior to CRRT initiation is a strong predictor of survival in sepsis-associated AKI patients [26]. In our study, initial 6 hr urine output at ER admission was found to be a quick and easy predictive tool for 30-day and 90-day mortality in patients with AKI undergoing CRRT. Additionally, we observed that patients in the LUO group tended to receive CRRT earlier compared to those in the HUO group. However, the time of CRRT initiation itself was not significantly associated with either 30-day or 90-day mortality. This suggests that while urine output may influence clinicians’ decisions regarding CRRT timing, the prognostic value lies more in the urine output itself than in when CRRT is started. Although our study also found that SOFA score was significantly associated with 30-day and 90-day mortality, SOFA requires the integration of multiple physiological variables and laboratory parameters, which may be impractical in the urgent setting of the emergency room. In contrast, urine output can be easily and promptly measured at the bedside without the need for complex calculations, making it a highly feasible and intuitive prognostic marker in acute clinical decision-making environments where time is critical. Therefore, our findings emphasize that initial urine output at ER admission may serve as a valuable guide for early triage and decision-making for critically ill patients requiring CRRT. In our study, RRT-free days through day 30 and day 90 were related with initial 6 hr urine output at ER admission. These results mean that initial 6 hr urine output at ER admission is one of the important predictive factors for renal recovery.

Plasma NGAL is an excellent marker for the early diagnosis of AKI. Duda et al. [27] found that plasma NGAL can be a valuable predictive marker of mortality in critically ill patients. Previous studies have analyzed predictive mortality factors influencing plasma NGAL levels in critically ill patients with AKI [28]. In an observational study of acute kidney injury carried out in Finland, urine NGAL was found to be a biomarker for critically ill patients with sepsis [29]. Our study demonstrated no correlation between plasma NGAL level and 30-day and 90-day mortality in patients undergoing CRRT. A possible explanation for why NGAL was not associated with 30-day and 90-day mortality in our study is heterogenicity of the causes of critically ill patients, including sepsis, GI bleeding, and cardiogenic shock. In our study, an infectious cause (sepsis) of mortality was related to a high NGAL level, and those cases involving a non-infectious cause of mortality, such as GI bleeding or cardiogenic shock, demonstrated relatively low NGAL levels (Figure S3). The previous literature supports the observation that NGAL behaves differently across various clinical settings. In patients with cardiovascular disease, plasma NGAL has been shown to predict mortality and readmission, with cutoff values around 192–227 ng/mL [30,31]. In sepsis, plasma NGAL levels above 387 ng/mL have demonstrated high sensitivity (81.0%) and specificity (67.8%) for predicting 28-day mortality [32]. In contrast, in patients with hemorrhagic shock due to abdominal trauma, plasma NGAL levels were significantly lower, with median values of 115.9 ng/mL in the shock group compared to 58.5 ng/mL in controls, indicating more limited prognostic capacity [33]. These findings highlight the variability in NGAL cutoff thresholds and prognostic relevance depending on the underlying pathology. Therefore, in critically ill populations with mixed etiologies, a single NGAL value may not have universal prognostic applicability. Although our study reflected these trends, we were unable to perform further stratified analyses due to the limited number of patients in each diagnostic category. Larger, multicenter studies are needed to validate NGAL’s prognostic utility across diverse clinical conditions.

In many traditional studies, in the general population, a decline in eGFR was significantly associated with increased risk [34]. Haas et al. [35] showed a strong association between eGFR at the time of admission and 30-day mortality, percentage of ICU admissions, and longer hospital stay. This was classified into six categories: eGFR > 90 mL/min/1.73 m^2^, 60–89 mL/min/1.73 m^2^, 45–59 mL/min/1.73 m^2^, 30–44 mL/min/1.73 m^2^, 15–29 mL/min/1.73 m^2^, and <15 mL/min/1.73 m^2^. We divided our patients into three groups (eGFR < 15 mL/min/1.73 m^2^, <30 mL/min/1.73 m^2^, and <60 mL/min/1.73 m^2^) and found no statistical correlation between 30-day and 90-day mortality and initial eGFR at the ER. However, an initial eGFR above 30 mL/min/1.73 m^2^ is a predictive marker for renal recovery.

Our study had several strengths. Compared with previous studies, we presented faster and easier predictive tools, especially initial 6 hr urine volume, for critically ill patients’ survival at ER. Commonly used survival scoring systems, such as APACHE II, SOFA, and SAPS, are more complex and time-consuming. Further, RRT-free duration, which shows the functioning of renal recovery, is associated with initial 6 hr urine volume at the ER.

Our study has some limitations. First, this is a single-center retrospective chart review study with a limited sample size. Second, due to the retrospective design, detailed information on the dosage and timing of diuretic administration during the first 6 h in the emergency room was not consistently available. As a result, we were unable to adjust for this potentially confounding variable in our analysis. Third, other volume status metrics, such as inferior vena cava diameter and central venous pressure, were not available for comparison. Fourth, we did not evaluate the time interval between the initial ER clinical factor and the initiation of CRRT. Fifth, Although the decision to initiate CRRT is generally made to address complications in renal failure in hemodynamically unstable situations, the timing and initiation of CRRT can vary depending on the individual patient’s condition and are determined by the attending nephrologist. In this study, the initiation of CRRT was decided by four nephrologists working at our institution. This variability in clinical judgment may have introduced differences in treatment timing among patients, representing a potential limitation of the study. Finally, future studies should employ larger cohorts and a prospective study design.

## 5. Conclusions

Our study demonstrated that the initial 6-h urine output measured at emergency room (ER) admission is significantly associated with both 30-day and 90-day mortality in critically ill patients who are subsequently admitted to the ICU and receive continuous renal replacement therapy (CRRT). Furthermore, early urine output was also associated with the number of RRT-free days at both time points. These findings suggest that assessing urine output during the first 6 h after ER arrival may serve as a simple and valuable prognostic indicator in the early triage and management of patients requiring CRRT.

## Figures and Tables

**Table 1:** Baseline characteristics.

Characteristic	Low Urine Output (*n* = 124)	High Urine Output (*n* = 66)	Total (*n* = 190)	*p*
Demographic data				
Age, mean (SD) (yr)	66.8 ± 15.2	70.2 ± 11.6	68.0 ± 14.1	0.094
Sex (male, %)	81 (65.3)	39 (59.1)	120 (63.1)	
SBP (mmHg)	94.5 ± 28.5	120.3 ± 36.0	103.5 ± 33.6	<0.001
DBP (mmHg)	55.3 ± 18.0	67.1 ± 18.3	59.4 ± 18.9	<0.001
BMI (kg/m^2^)	21.45 ± 7.22	22.39 ± 7.05	21.78 ± 7.15	0.392
Laboratory parameters				
Hemoglobin (g/dL)	11.07 ± 3.12	11.40 ± 2.59	11.18 ± 2.94	0.464
Glucose (mg/dL)	209.77 ± 177.69	208.39 ± 148.48	209.29 ± 168.10	0.947
Serum albumin (g/dL)	2.83 ± 0.63	2.98 ± 0.60	2.89 ± 0.62	0.104
Lactic acid (mmol/L)	7.80 ± 5.18	5.07 ± 4.54	6.85 ± 5.12	<0.001
CRP (mg/dL)	9.29 ± 10.69	10.00 ± 11.34	9.53 ± 10.89	0.666
NGAL (ng/mL)	1066.91 ± 1006.39	511.34 ± 598.10	873.92 ± 923.32	<0.001
BUN (mg/dL)	47.73 ± 29.52	36.95 ± 25.50	43.98 ± 28.59	0.010
Serum creatinine (mg/dL)	3.23 ± 2.38	2.10 ± 1.52	3.29 ± 5.81	<0.001
eGFR (mL/min/1.73 m^2^)	30.52 ± 24.95	44.94 ± 33.96	35.53 ± 29.14	0.001
Potassium (mEq/L)	4.73 ± 1.18	4.42 ± 1.09	4.62 ± 1.16	0.075
Bicarbonate (mEq/L)	15.04 ± 6.82	18.06 ± 6.45	16.09 ± 6.83	0.003
UPCR (mg/mg)	1.45 ± 1.18	1.44 ± 1.18	1.45 ± 1.18	0.075
Urine hematuria (%)	66 (53)	29 (44)	95 (47.8)	0.003
FV (mL)	490.7 ± 294.2	525.2 ± 342.2	502.7 ± 311.2	0.460
UV (mL)	60.3 ± 54.6	653.7 ± 390.3	266.4 ± 366.9	<0.001
SOFA score	7.47 ± 2.47	6.61 ± 2.55	7.17 ± 2.53	0.027
CRRT start (h) *	14.32 ± 3.79	17.35 ± 5.5	15.37 ± 4.67	<0.001
Cause of CRRT (%)				
Septic shock (%)	53 (43)	38 (57)	91 (48)	0.051
** Cardiologic problem (%)	30 (24)	12 (18)	42 (22)	0.036
*** Hypovolemic problem (%)	17 (14)	3 (0.5)	20 (11)	0.050
**** Others (%)	24 (19)	13 (20)	37 (19)	0.955
Comorbidities (%)				
Hypertension (%)	73 (58.9)	39 (59.1)	112 (58.9)	0.217
Diabetes mellitus(%)	58 (46.8)	40 (60.6)	98 (51.6)	0.069
Heart failure (%)	17 (13.7)	8 (12.1)	25 (13.2)	0.758
Cardiovascular disease (%)	15 (12.1)	6 (9.1)	21 (11.1)	0.529

Abbreviations: SBP, systolic blood pressure; DBP, diastolic blood pressure; BMI, body mass index; SOFA, Sequential Organ Failure Assessment; CRRT, continuous renal replacement therapy; NGAL, neutrophil gelatinase-associated lipocalin; CRP, C-reactive protein; BUN, blood urea nitrogen; eGFR, estimated glomerular filtration rate; UPCR, urine protein creatinine ratio; FV, volume of input fluid; UV, urine volume for 6 h. * CRRT start (h): The time from emergency room admission to the initiation of CRRT. ** Cardiologic problem: heart failure, ischemic heart disease, arrythmia. *** Hypovolemic problem: gastrointestinal bleeding, postpartum bleeding, heat stroke. **** Others: hemodynamic unstable status caused by metabolic acidosis due to alcohol, drugs, or brain hemorrhage such as subarachnoid hemorrhage.

**Table 2:** Hazard ratio (HR), 95% CI, and *p*-value for factors determined from multivariable Cox regression analysis of risk factors for 30-day mortality and 90-day mortality.

	30-Day Mortality			90-Day Mortality		
Significant Variable	HR	95% CI	*p*	HR	95% CI	*p*
Urine < 0.5 mL/kg/h *	1.88	1.10–3.25	0.023	2.07	1.26–3.42	0.004
SBP (mmHg)	1.01	0.99–1.02	0.453	1.01	1.00–1.02	0.143
DBP (mmHg)	0.98	0.96–1.00	0.101	0.98	0.96–1.00	0.053
NGAL (ng/mL)	1.00	1.00–1.00	0.755	1.00	1.00–1.00	0.604
eGFR (mL/min/1.73 m^2^)	0.99	0.98–1.01	0.293	0.99	0.98–1.00	0.138
BUN (mg/dL)	1.00	0.98–1.01	0.513	0.99	0.98–10.1	0.292
Creatinine (mg/dL)	0.91	0.76–1.10	0.324	0.92	0.78–1.10	0.351
Albumin (g/dL)	0.79	0.56–1.17	0.236	0.74	0.51–1.06	0.097
HCO_3_ (mEq/L)	0.98	0.95–1.02	0.346	0.98	0.95–1.02	0.313
SOFA score	1.26	1.09–1.46	0.002	1.21	1.07–1.37	0.003

Abbreviations: SBP, systolic blood pressure; DBP, diastolic blood pressure; NGAL, neutrophil gelatinase-associated lipocalin; eGFR, estimated glomerular filtration rate; BUN, blood urea nitrogen. * Average urine volume of <0.5 mL/kg/h over 6 h.

**Table 3:** Relation between RRT-free days and clinical parameters.

	LUO (*n* = 124)	HUO (*n* = 66)	Mean Difference (95% CI)	*p*
RRT-free days through day 30 mean ± SD	7.6 ± 12.1	15.1 ± 14.0	−7.33 (−11.36 to −3.29)	0.000
RRT-free days through day 90 mean ± SD	27.0 ± 39.0	47.1 ± 42.5		0.002
	eGFR < 15 mL/min/1.73 m^2^ (*n* = 42)	eGFR ≥ 15 mL/min/1.73 m^2^ (*n* = 148)	Mean Difference (95% CI)	*p*
RRT-free days through day 30 mean ± SD	7.1 ± 11.4	11.2± 13.6	−4.05 (−8.20 to 0.11)	0.056
RRT-free days through day 90 mean ± SD	25.9 ± 38.1	36.3± 41.9	−10.41 (−23.99 to 3.17)	0.131
	eGFR < 30 mL/min/1.73 m^2^ (*n* = 98)	eGFR ≥ 30 mL/min/1.73 m^2^ (*n* = 92)	Mean Difference (95% CI)	*p*
RRT-free days through day 30 mean ± SD	7.6 ± 11.9	13.1 ± 14.0	−5.50 (−9.23 to −1.77)	0.004
RRT-free days through day 90 mean ± SD	26.2 ± 38.3	42.3 ± 42.8	−16.14 (−27.80 to −4.49)	0.007
	eGFR < 60 mL/min/1.73 m^2^ (*n* = 165)	eGFR ≥ 60 mL/min/1.73 m^2^ (*n* = 25)	Mean Difference (95% CI)	*p*
RRT-free days through day 30 mean ± SD	9.5 ± 12.9	15.8 ± 14.4	−6.39 (−12.61 to −0.16)	0.045
RRT-free days through day 90 mean ± SD	31.7 ± 40.6	49.4 ± 43.0	−17.75 (−35.06 to −0.43)	0.045
	NGAL < 364 ng/mL (*n* = 76)	NGAL ≥ 364 ng/mL (*n* = 114)	Mean Difference (95% CI)	*p*
RRT-free days through day 30 mean ± SD	11.7 ± 14.0	9.3 ± 12.7	2.38 (−1.56 to 6.32)	0.234
RRT-free days through day 90 mean ± SD	36.6 ± 42.6	32.3 ± 40.4	4.36 (−7.70 to 16.42)	0.477

Abbreviations: eGFR, estimated glomerular filtration rate; NGAL, neutrophil gelatinase-associated lipocalin.

## Data Availability

The original contributions presented in the study are included in the article/Supplementary Material; further inquiries can be directed to the corresponding authors.

## Supplementary Materials

The following supporting information can be downloaded at: https://www.mdpi.com/article/10.3390/life15060866/s1, Figure S1: Kaplan–Meier curves for cumulative probability of 30-day; Figure S2: Kaplan–Meier curves for cumulative probability of 90-day; Figure S3: Box plots showing median plasma NGAL between non-infectious cause of death and infectious cause of death.
