# Supplementary material for: Early Urine Output in the Emergency Room as a Prognostic Indicator for Critically Ill Patients Undergoing Continuous Renal Replacement
Source: Life (Basel). 2025 May 27;15(6):866. doi: 10.3390/life15060866 (PMC12194583; doi:10.3390/life15060866)

Supplementary Materials

Supplementary Figure S1. Kaplan-Meier curves for cumulative probability of 30-day.
We divided groups by initial eGFR 15 ml/min1.73m2, 30 ml/min/1.73m2, 60 ml/min/1.73m2 and there was not significantly difference (*P*= 0.999, *P*= 0.615, and *P*= 0.405). By plasma NGAL 364 ng/mL level, there was no significantly different mortality (*P*= 0.814).

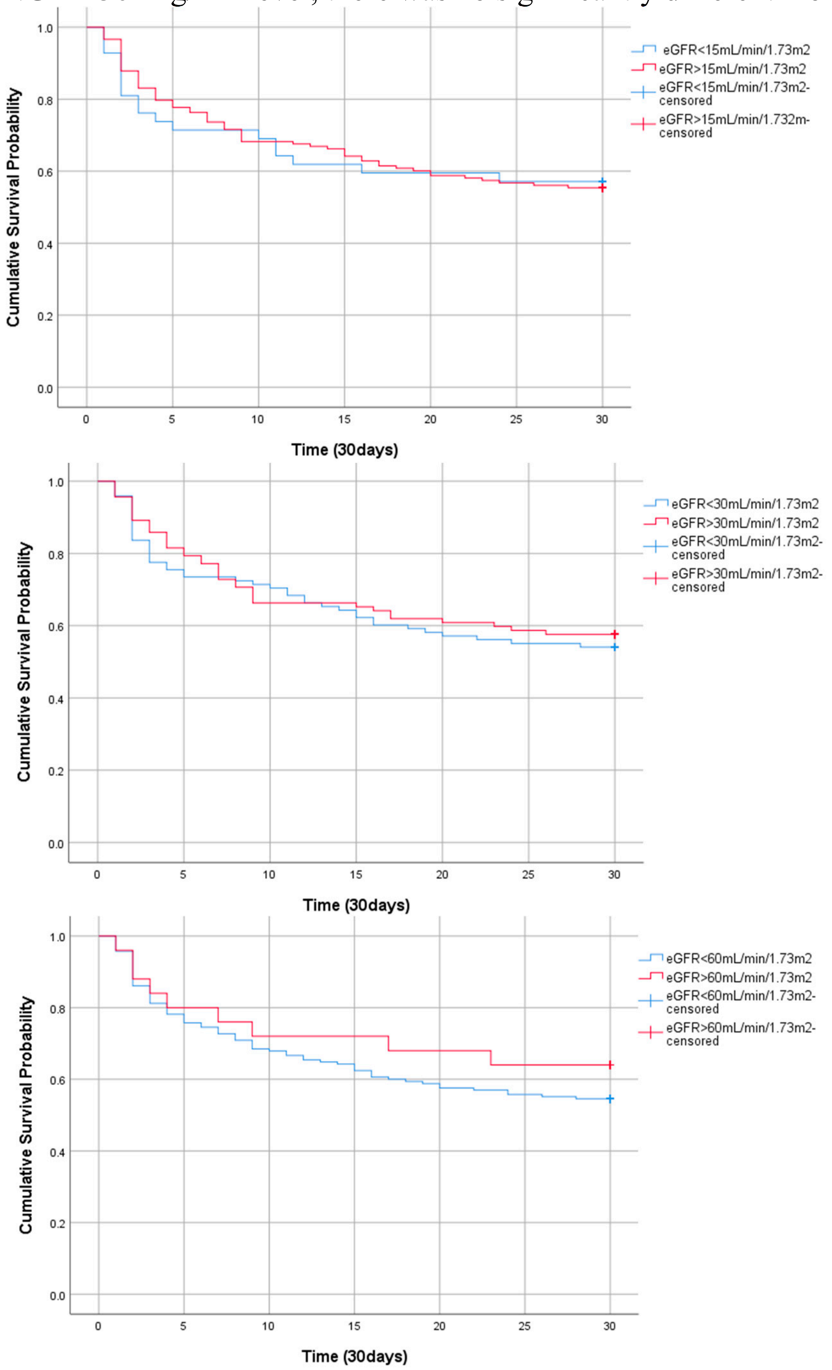

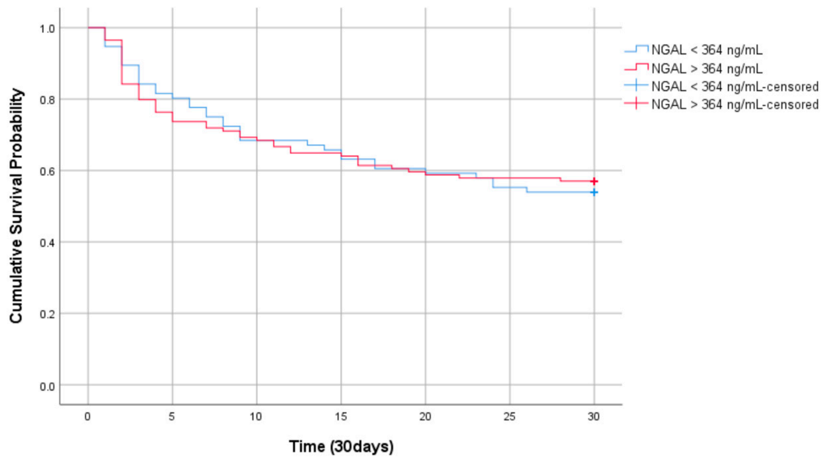

eGFR<15ml/min/1.73m2 (*P*= 0.999)
eGFR<30ml/min/1.73m2 (*P*= 0.615)
eGFR<60ml/min/1.73m2 (*P*= 0.405)
NGAL 364 ng/mL (*P*= 0.814)

Supplementary Figure S2. Kaplan-Meier curves for cumulative probability of 90-day. We divided groups by initial eGFR 15 ml/min1.73m2, 30 ml/min/1.73m2, 60 ml/min/1.73m2 and there was not significantly difference (*P*= 0.756, *P*= 0.247, and *P*= 0.372). By plasma NGAL 364 ng/mL level, there was no significantly different mortality (*P*= 0.937).

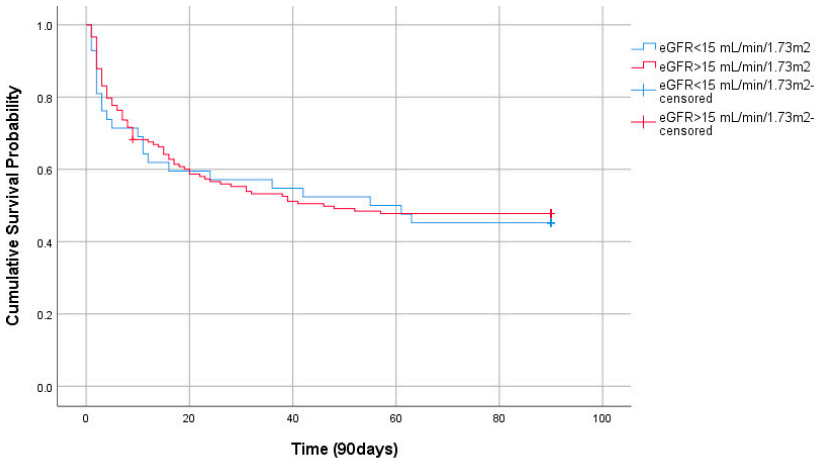

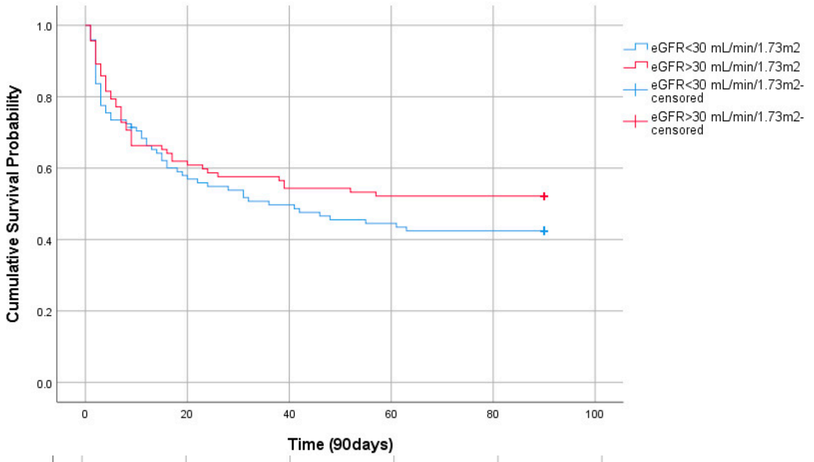

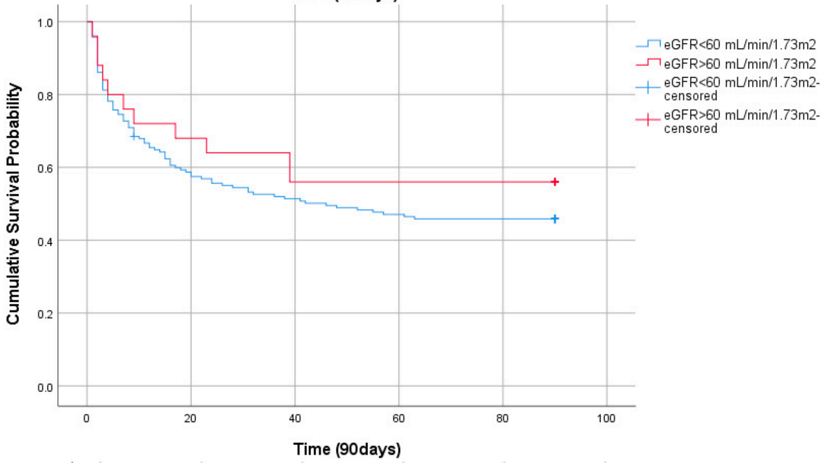

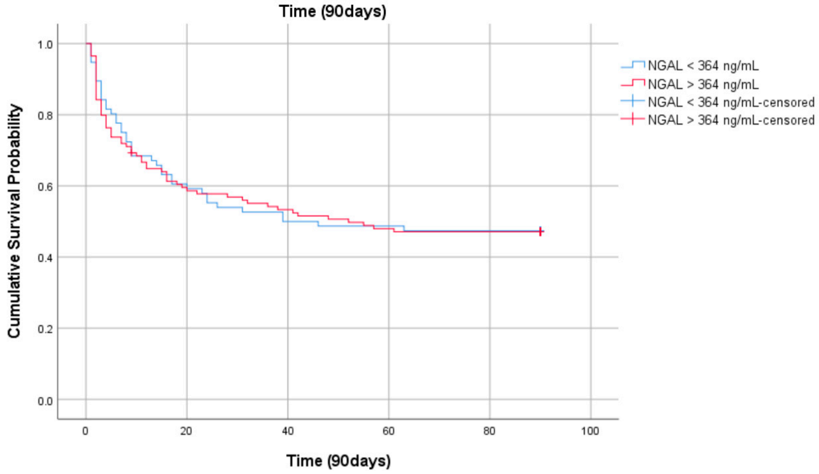

eGFR<15ml/min/1.73m2 (*P*= 0.756)
eGFR<30ml/min/1.73m2 (*P*= 0.247)
eGFR<60ml/min/1.73m2 (*P*= 0.372)
NGAL 364 ng/mL (*P*= 0.937)

Supplementary Figure S3. Box plots showing median plasma NGAL between non-infectious cause of death and infectious cause of death. Medan plasma NGAL level was 345.53 ng/mL in non-infectious cause of death group and 1499.50 ng/mL in infectious cause of death group ($P < 0.001$).

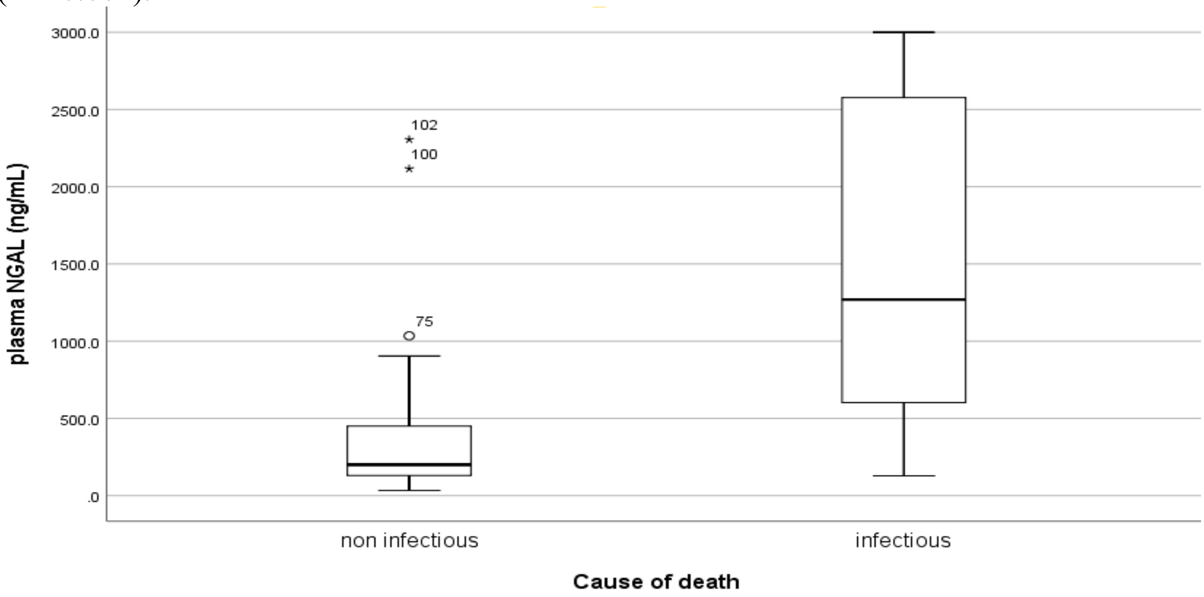

Supplement: Supplementary file 1 [file life-15-00866-s001.zip › life-3570597-supplementary.pdf]
